# Gravid females of the mosquito *Aedes aegypti* avoid oviposition on *m*-cresol in the presence of the deterrent isomer *p*-cresol

**DOI:** 10.1186/1756-3305-7-315

**Published:** 2014-07-09

**Authors:** Ali Afify, C Giovanni Galizia

**Affiliations:** 1 Neurobiology, University of Konstanz, 78457 Konstanz, Germany

**Keywords:** Mosquitoes, Odor, Egg laying, Deterrence

## Abstract

**Background:**

*p*-cresol (4-methylphenol) and its isomer *m*-cresol (3-methylphenol) have been shown to activate the same sensilla in *Aedes aegypti* (Linnaeus) mosquitoes. Whereas *p*-cresol has been suggested to play a role in oviposition site choice, the behavioral significance of *m*-cresol is unknown.

**Methods:**

Here, we assayed the oviposition behavior of *Aedes aegypti* towards *p*-cresol and *m*-cresol using cage assay. Specifically we tested different concentrations of *p*-cresol (10^-12^-10^3^ ppm) and *m*-cresol (10^-1^-10^3^ ppm), the 1:1 mixture of the two compounds at 10^2^ ppm, and the two individual compounds at 10^2^ ppm together in the same cage.

**Results:**

We show that (1) *p*-cresol is a stimulant at a low concentration and deterrent over a broad range of higher concentrations (10^-8^-10^3^ ppm), while *m*-cresol was behaviorally ineffective, except for a deterrent effect at the highest concentration (10^3^ ppm) (2) in concentration choice tests (different concentrations tested against each other), both compounds were deterrent only at the highest concentration (3) a 1:1 mixture of both compounds exhibited a deterrent effect on oviposition (4) when presented in separate cups but together in the same cage, *p*-cresol and *m*-cresol (10^2^ ppm) both received significantly less eggs than water alone.

**Conclusions:**

Our results suggest that *p*-cresol is a strong oviposition deterrent with a stimulant effect at only a very low concentration, while *m*-cresol is not a deterrent *per se.* However, in the presence of *p*-cresol in the vicinity, *m*-cresol acts as a deterrent. This finding adds a new twist to the possible interactions of different odors in oviposition site choice: not only the source itself, but nearby odors also influence a mosquito’s choice.

## Background

With a relatively short life cycle and a limited number of oviposition events, choosing a substrate for oviposition is a critical decision for mosquitoes. Mosquitoes depend on olfactory cues to locate their oviposition sites, in addition to other cues (visual, tactile) [1,2] and also weather patterns that could affect the concentration of olfactory cues in the oviposition substrates [3,4]. Odors of oviposition substrates may carry information about food availability [5,6], the presence of conspecific larvae [7,8], or predators [9], and thus play a critical role in choosing a suitable oviposition site for the offspring. For example, mosquito larvae feed on microorganisms that develop on plant detritus in the water, and the type of detritus affects growth and survival of the larvae [10-12]. Gravid females that are attracted and/or stimulated to lay eggs by the smell of plant infusions might use this smell as an indicator for the quality of food resources at that site [13-15].

A clear terminology was proposed to describe olfactory cues that affect mosquito oviposition decision [16]; an “oviposition attractant” is a substance that encourages gravid females to make oriented flights towards the oviposition substrate while a “stimulant” is a substance that elicits oviposition. Also, a “repellent” is a substance that encourages an oriented flight away from the oviposition substrate while a “deterrent” is a substance that inhibits oviposition. Here, we follow this terminology.

**Figure 1:**
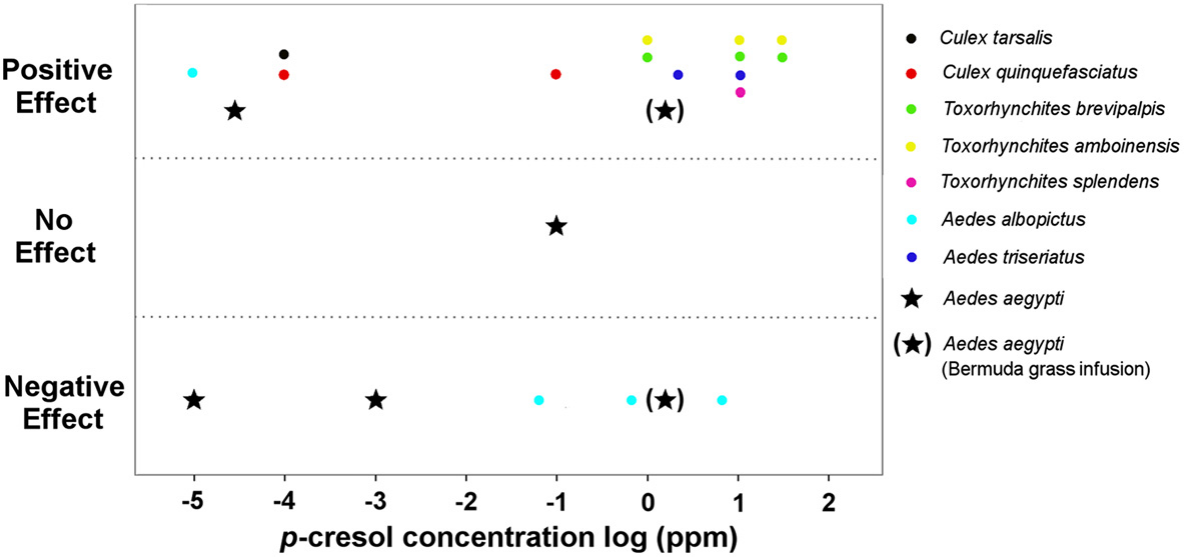
**A summary of literature data shows the published effects of *p*-cresol on different mosquito species. ***p*-cresol has different effects (positive, negative and no effect) on various mosquito species. Data are contradictory for the effect of *p*-cresol on *Ae. aegypti* at 10^-5^-10^-3^ ppm concentration range. The effect of Bermuda grass infusion on *Ae. aegypti* is also plotted (at 1.99 ppm *p*-cresol). Data from: [17-27].

*p*-cresol is a key volatile component present at a concentration of 1.99 mg/liter (1.99 ppm) in crude extract of Bermuda grass infusion [17]. Bermuda infusions were shown to either stimulate [18] or to deter/repel [19,20] oviposition of *Ae. aegypti* gravid females (Figure 1). *p*-cresol alone at a concentration of 0.01 or of 1.0 μg/liter (10^-5^ or 10^-3^ ppm) deterred oviposition in *Ae. aegypti*, but this deterrent effect disappeared at 10^-1^ ppm [19] (Figure 1). In contrast, *p*-cresol was found to be a stimulant in another study, where 20 μl 10^-4^ solution was applied on a filter paper which was afterwards half submerged in a 50 ml volume of water, resulting in a dilution of nominally 0.04 μl/l, i.e. 4*10^-5^ ppm [21].

*p*-cresol was also tested in other species (Figure 1): it is an oviposition attractant for *Aedes triseriatus* at 3 and 10 ppm [22,23]. In addition, it attracted gravid *Culex quinquefasciatus* and *Culex tarsalis* at 0.1 μg/liter (10^-4^ ppm) [24]. *p*-cresol also stimulated egg laying of *Cx. quinquefasciatus* at a 100 μg/liter (10^-1^ ppm) [25]. *p*-cresol stimulated oviposition in two species of *Toxorhynchites* mosquitoes (*Tx. brevipalpis* and *Tx. amboinensis*) at concentrations of 1, 10 and 50 ppm while it stimulated oviposition of *Tx. splendens* at 10 ppm [26]. *p*-cresol stimulated *Aedes albopictus* oviposition at 0.01 μg/liter (10^-5^ ppm) [19]. In a separate study, three concentrations of *p*-cresol (0.083, 0.83 and 8.3 mg/liter) were repellent against *Ae. albopictus* gravid females, with the greatest effect at 8.3 mg/liter (8.3 ppm), suggesting that *p*-cresol acts as a deterrent for several mosquito species [27].

Thus, *p*-cresol elicited a wide range of responses with different mosquito species (Summarized in Figure 1), with a negative effect only on *Ae. albopictus* and *Ae. aegypti*. In addition, reports about the response of *Ae. aegypti* are contradictory for similar concentrations of *p*-cresol; deterrent at 10^-5^ and 10^-3^ but stimulant at 4*10^-5^ ppm. We therefore sought to reexamine the effect of *p*-cresol on *Ae. aegypti* oviposition over a wide range of concentrations under unified experimental conditions.

In behavioral studies, the isomer *m*-cresol stimulated oviposition of *Ae. triseriatus* at 3 ppm [23] and stimulated/attracted oviposition of gravid *Toxorhynchites moctezuma* and *Toxorhynchites amboinensis* mosquitoes [28]. For *Ae. aegypti,* Siju *et al. *[29] measured the responses of sensilla trichodea in females against *p*-cresol and *m*-cresol across the gonotrophic cycle using single sensillum recordings. Some receptor cell types showed similar responses for both odorants, and the sensitivity to both odorants increased after blood feeding in some of the short blunt tipped II trichoid sensilla, suggesting that these substances might be perceived similarly by the female mosquito and that also *m*-cresol might have a role in oviposition [29]. However, *m*-cresol has not yet been tested behaviorally against *Ae. aegypti* at any concentration; it is not known whether the similarity in structure and receptor cell response towards the two isomers would result in a similar deterrent effect of the two isomers. Equally unknown is the effect of the two compounds presented together or in a mixture.

Therefore, in this study, we used a laboratory bioassay to test the oviposition behavior of *Ae. aegypti* towards differing concentrations of *p*-cresol (10^-12^-10^3^ ppm) and *m*-cresol (10^-1^-10^3^ ppm), the 1:1 mixture of the two compounds at 10^2^ ppm, and the two individual compounds at 10^2^ ppm together in the same cage.

## Methods

### Mosquito colony

*Ae. aegypti* eggs were obtained from Biogents AG (Regensburg, Germany). After hatching, mosquito larvae were fed on fish food (TetraMin®, Tetra GmbH, Melle, Germany) every other day. Cotton pads soaked with sugar solution (10%, w/vol) were provided to feed adult mosquitoes as a source of carbohydrates. Mosquito females were blood fed on pigeons for egg laying. Mosquitoes were raised and all experiments were done in a climate chamber maintained at a 25–28°C temperature, 60–70% relative humidity and L12:D12 photoperiod. The climate chamber was in complete darkness during the dark cycle (between 7 pm and 7 am). The use of pigeons in blood feeding was done at the animal research facility of the university of Konstanz and approved by the authorities according to German law (TierSchG §10a, approval 35–9185.82/I).

### Bioassay

**Figure 2:**
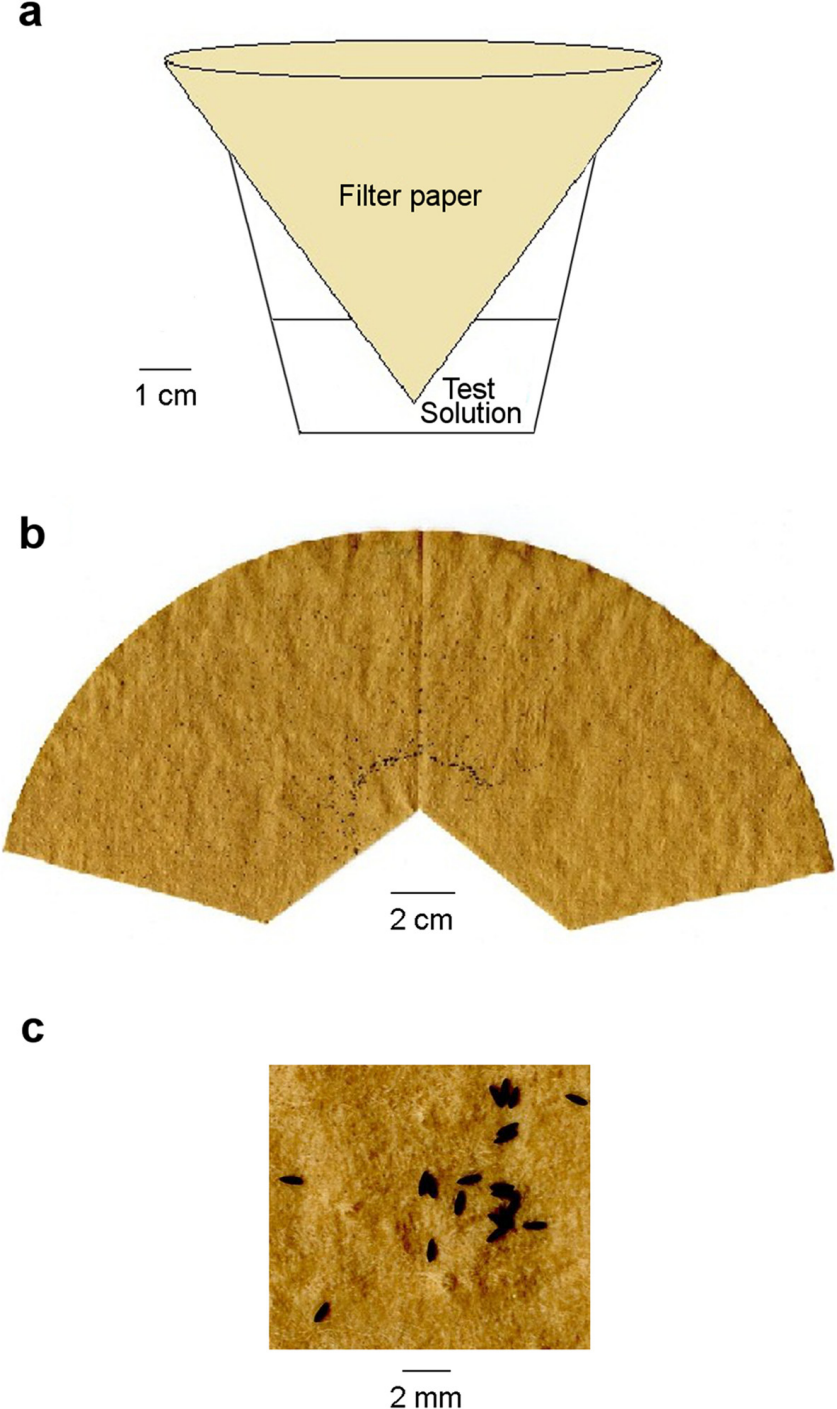
**Oviposition cup and the filter paper. a)** Oviposition cup with a filter paper immersed in the test solution, the bottom edge of the filter paper is cut and closed in a pointed shape. **b)** Opened filter paper with *Ae. aegypti* eggs ready for scanning. **c)** A piece of the filter paper with high magnification showing the individual eggs.

Oviposition response was tested following previously described bioassay [5,30] with some modifications. Experiments were done in white plastic mosquito boxes (30 × 30 × 30 cm) with three mesh sides. On the day of the experiment, each box was provided with 20 blood fed females (1–2 week old, four days post blood feeding) and the oviposition cups: two cups for each stimulus when testing one odorant or the mixture against water, or one cup of each odorant when testing more than one odorant/concentration in the same cage. In all experiments, oviposition cups were placed pseudorandomly at fixed positions in the corners of the cage. We also tested whether the gravid females have an initial preference towards any of the four positions. A “non-choice” experiment was done in which the mosquitoes were offered four cups of clean water. Mosquitoes distributed the eggs equally in the four cups (ANOVA, P = 0.8, n = 5) showing no position bias between the different corners of the cage.Oviposition cups were white plastic cups (181 ml) that contained 30 ml of the test solution and a piece of brown coffee filter paper (Melitta®, Minden, Germany). Filter paper was not treated before the experiments, thus any potential background odor from the paper was equal throughout all experiments. The bottom edge of the filter paper was cut and then twisted into a pointed closed edge to prevent mosquitoes from laying eggs outside the filter paper. Then the filter paper was partially immersed in the stimulus solution (Figure 2a). Experiments started at 3–4 pm and were stopped at 10 am the next morning. Plastic cups were discarded after one use. The total number of eggs on each filter paper was counted (Figure 2b, c).

**Figure 3:**
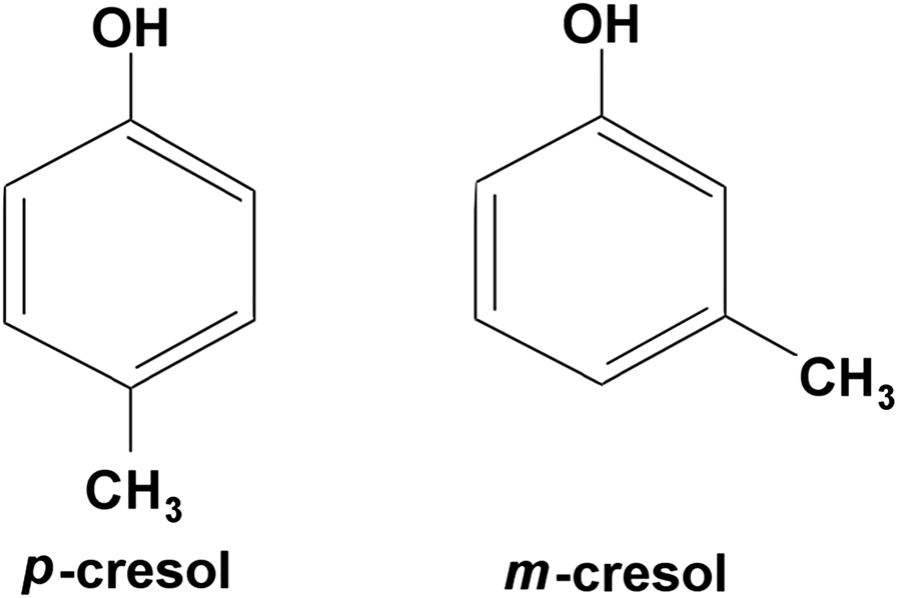
**Chemical structure of *p*-cresol and *m*-cresol.**

We tested *p*-cresol and its isomer *m*-cresol (Figure 3). We prepared stock solutions of *p*-cresol (SAFC, St. Louis, USA. ≥98% purity) and *m*-cresol (Sigma Aldrich, St. Louis, USA. 99% purity) in *n*-hexane (Fisher, Loughborough, UK. 99% purity) and added 1 ml of each solution to 30 ml of water to reach the indicated final concentration. We also added 1 ml of *n*-hexane to the 30 ml of water in control cups. We allowed *n-*hexane to evaporate for 30 min before adding the filter papers. Control experiments showed that *n-*hexane had completely evaporated after this period of 30 min (data not shown).

Odorants were tested in the following conditions:

1- A range of decreasing concentrations of *p*-cresol (10^3^-10^-12^ ppm).

2- A range of decreasing concentrations of *m*-cresol (10^3^-10^-1^ ppm). We did not test beyond 10^-1^ ppm because the range 10^2^-10^-1^ ppm did not elicit any behavioral effect.

3- Series of three consecutive concentrations in one cage (either 10^-1^, 10°, 10^1^; or 10^1^, 10^2^, 10^3^ ppm) tested against water, for both isomers.

4- A 1:1 mixture of both compounds at 10^2^ ppm (50 ppm of each compound) against water.

5- *p*-cresol and *m*-cresol compared to each other at 10^2^ ppm (together in the same cage against water) to test for interactions between the two compounds.

All experiments were performed separately in a closed climate chamber, and only one odor was tested at a time (except when testing interactions) to reduce the effect of background odors.

### Analysis

Filter papers were opened (Figure 2b), dried, scanned using an Epson perfection 1670 scanner (Seiko Epson Corporation, Suwa, Nagano, Japan) and the photos were then analyzed using ImageJ [31] for egg numbers.

First, images of egg papers were converted into 8 bit images, and then the “Threshold” function in ImageJ was used to select the dark areas in the image. A threshold was set to select the area of all eggs without selecting other areas in the image. The “Analyze particles” function was then used twice to:

1- Calculate the total area of all eggs. The function was set to calculate the area of all particles from 40 pixels (minimum area of an egg) to infinity.

2- Calculate the average area of an egg. The function was set to calculate the average area of all particles from 40 pixels (minimum area of an egg) to 70 pixels (maximum area of an egg).

Finally, the number of eggs on the filter paper was calculated as the ratio of the two readings of total area and average area of the individual egg:

eggnumber=totaleggarea/averageareaofoneegg

Automatic counting was verified by randomly selecting 10 egg papers and comparing the results with visual counts using a stereomicroscope. There was no significant difference between automatic and manual counting (291 ± 105 eggs in the automatic counts and 295 ± 115 in visual counts, mean ± SD, paired *t*-test, n = 10, P = 0.713).

For a comparison between the effect of different concentrations, we used the oviposition activity index (OAI) described by Kramer and Mulla [32]:

$$\mathrm{OAI}\;=\;\frac{\mathrm{NT}\;‒\;\mathrm{NS}}{\mathrm{NT}\;+\;\mathrm{NS}}$$

N_T_ = number of eggs laid on the test solution.

N_S_ = number of eggs laid on the control solution.

The OAI values fall within -1 and 1, where negative values indicate a deterrent effect and positive values indicate a stimulant effect.

The data of all experiments are discrete numbers, and eggs laid in one cup cannot be laid in other cups. However, egg numbers were high and their distribution was approximately normal. Therefore, we used parametric significance tests. Paired *t*-tests were used where appropriate. For multiple comparisons, two-way ANOVA with the factors “cup”, “cage” and “interaction” was used followed by pairwise *t*-tests with Holm correction. There was no significance for factors “cage” or “interaction” in any of the experiment, so we omit reporting their values. All analyses were conducted in R [33].

## Results

### Oviposition effect of *p*-cresol

**Figure 4:**
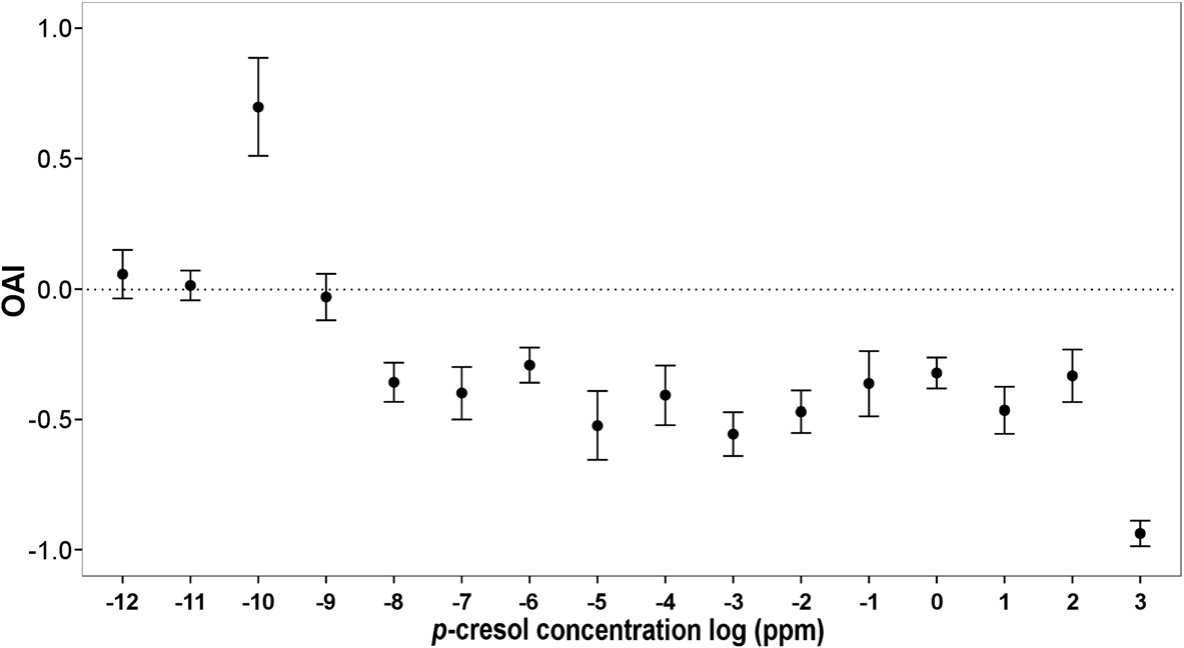
**Oviposition activity indices (OAI) of *p*-cresol at a broad range of concentrations. ***p*-cresol shows a dose dependent oviposition effect on *Ae. aegypti* gravid females, in which a low concentration (10^-10^ ppm) is stimulant while higher concentrations (10^-8^-10^3^ ppm) are deterrent. Each data point represents the mean OAI and standard error of five oviposition cages (n = 5) except for 10^2^ ppm (n = 13).

We tested the oviposition effect of *p*-cresol against water for decreasing decadic concentration steps 10^3^-10^-12^ ppm (Figure 4). In the upper concentration range (10^-8^-10^3^ ppm), *p*-cresol had a deterrent effect on *Ae. aegypti* oviposition (P = 0.012, 0.020, 0.016, 0.017, 0.025, 0.005, 0.006, 0.048, 0.008, 0.008, 0.009 and 0.003 for 10^-8^, 10^-7^, 10^-6^, 10^-5^,10^-4^, 10^-3^, 10^-2^, 10^-1^, 10°, 10^1^, 10^2^ and 10^3^ ppm *p*-cresol, respectively, individual paired *t-*tests). At 10^-9^ ppm *p-*cresol had no effect (P = 0.629), at 10^-10^ ppm it was a stimulant (P = 0.021), and when further diluting it was ineffective (P = 0.527 and 0.866 for 10^-12^ and 10^-11^ ppm, respectively). Thus, the effect of *p-*cresol onto oviposition was dose-dependent: deterrent at high concentrations, and stimulant at low concentration.

### Oviposition effect of *m*-cresol

**Figure 5:**
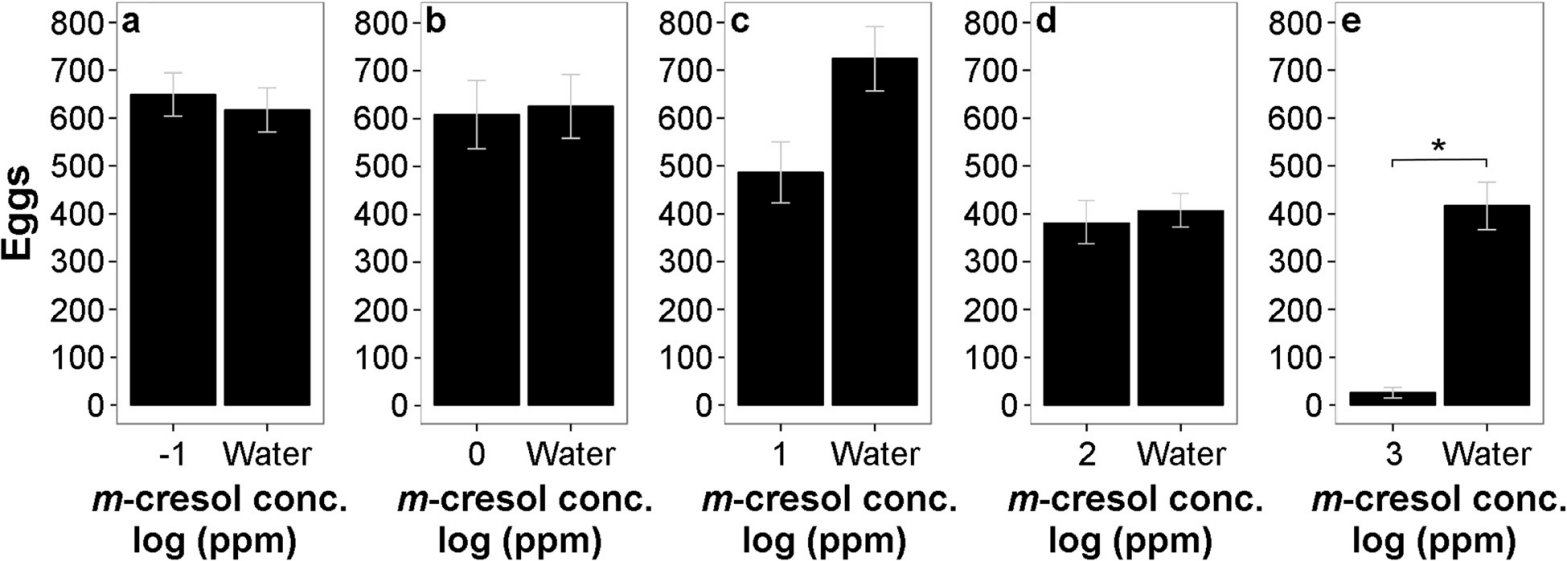
**Oviposition response of *Ae. aegypti *to different concentrations (individually tested) of *m*-cresol.** At the concentrations 10^-1^ ppm **(a)** to 10^3^ ppm **(e) ***m*-cresol shows a deterrent effect only at high concentrations; only 10^3^ ppm *m*-cresol received a statistically significant lower number of eggs than water (P = 0.003, paired *t-*test). (n = 5 for a, b, c, e; n = 13 for d). Asterisk indicates P < 0.05.

We tested the oviposition effect of *m*-cresol against water for decreasing decadic concentration steps 10^3^-10^-1^ ppm (Figure 5). At 10^3^ ppm *m*-cresol was highly deterrent (P = 0.003). At lower concentrations, *m*-cresol was not behaviorally active (P = 0.722, 0.906, 0.136 and 0.766 for 10^-1^, 10°, 10^1^, and 10^2^ ppm, respectively, individual paired *t-*tests). Given the lack of responses in this range, we did not test even lower concentrations.

### Comparative concentration effects for *p*-cresol and *m*-cresol

**Figure 6:**
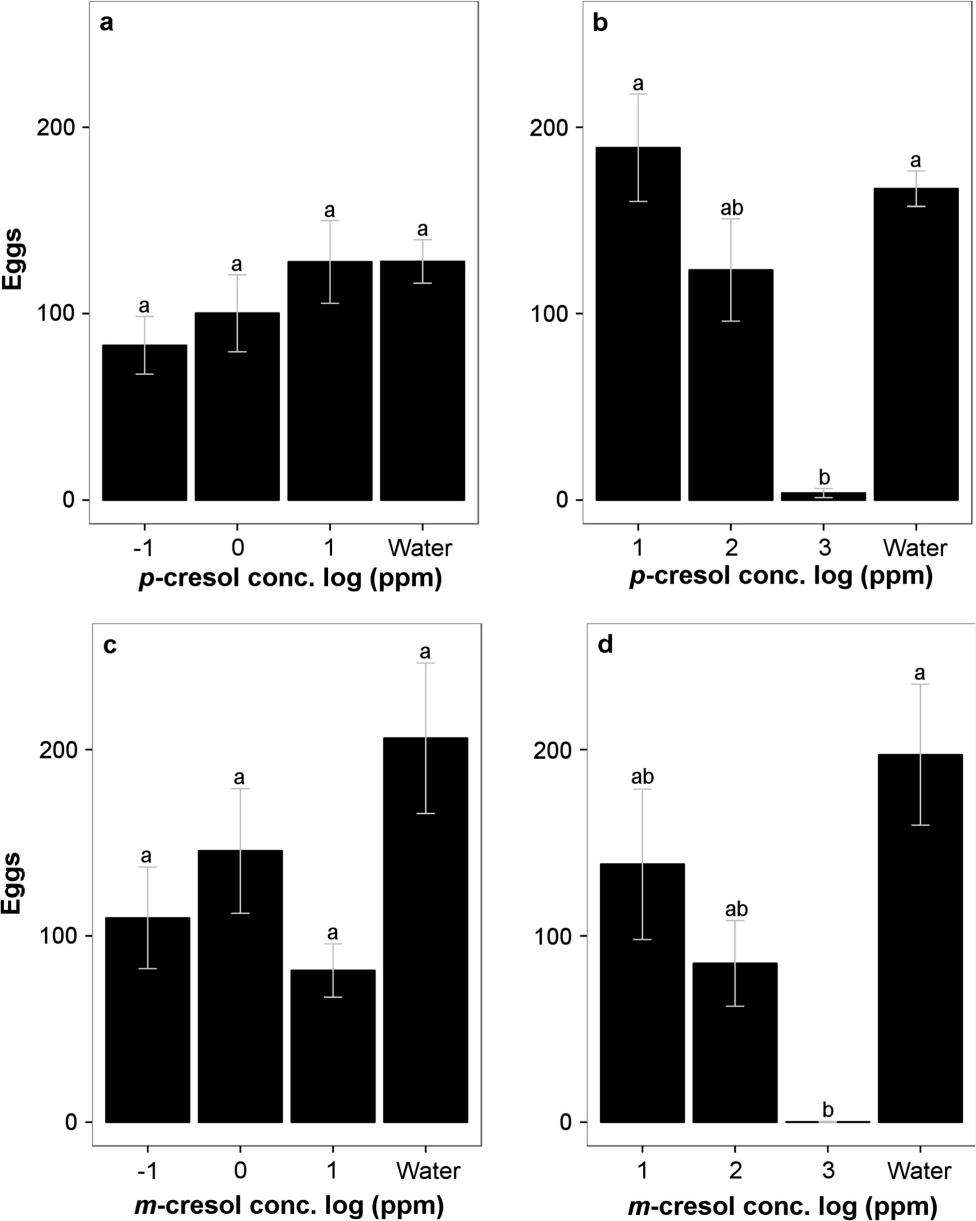
**Oviposition response of *Ae. aegypti *to different concentrations (concentration choice test) of *p*-cresol and *m*-cresol. a)** Response to water and 10^-1^ ppm, 1 ppm and 10 ppm of *p*-cresol were not statistically different (n = 5). **b)** Response to water and 10 ppm, 10^2^ ppm and 10^3^ ppm of *p*-cresol showed that increasing concentrations were increasingly deterrent (n = 5). **c)** Response to water and 10^-1^ ppm, 1 ppm and 10 ppm of *m*-cresol (n = 5) shows no deterrent effect of *m*-cresol. **d)** Response to water and 10 ppm, 10^2^ ppm and 10^3^ ppm of *m*-cresol (n = 5) shows a deterrent effect at high concentrations. Different letters indicate statistically significant differences. Pairwise *t*-test with Holm correction.

When given a choice of different concentrations, mosquitoes were deterred by cresols only at the highest concentration of 10^3^ ppm (Figure 6). Specifically, when presented with the choice of *p*-cresol at concentrations 10^-1^, 10°, 10^1^ ppm and water, none of the cups was deterrent (P = 0.222, two-way ANOVA, Figure 6a). Similarly, when presented with the choice of *m*-cresol, concentrations 10^-1^, 10°, 10^1^ ppm and water, none of the cups was deterrent (P = 0.115, two-way ANOVA, Figure 6c). However, when the cups offered 10^3^ ppm *p*-cresol, the group effect was highly significant (P < 0.001, two-way ANOVA): 10^3^ ppm was deterrent in comparison with water (P < 0.001, post hoc pairwise *t*-test with Holm correction) and also in comparison with 10^1^ ppm (P = 0.015, post hoc pairwise *t*-test with Holm correction), while 10^2^, 10^1^ ppm were not significantly different from water or each other (Figure 6b). Similarly, when the cups offered 10^3^ ppm *m*-cresol, the group effect was significant (P = 0.010, two-way ANOVA): 10^3^, 10^2^, 10^1^ ppm were not significantly different from each other in egg counts while only 10^3^ ppm was deterrent compared with water (P = 0.039, post hoc pairwise *t*-test with Holm correction). 10^2^ ppm *m*-cresol received a lower number of eggs than water but the difference was marginally not significant (P = 0.054, post hoc pairwise *t*-test with Holm correction, Figure 6d).

### Effect of mixing *m*-cresol and *p*-cresol

**Figure 7:**
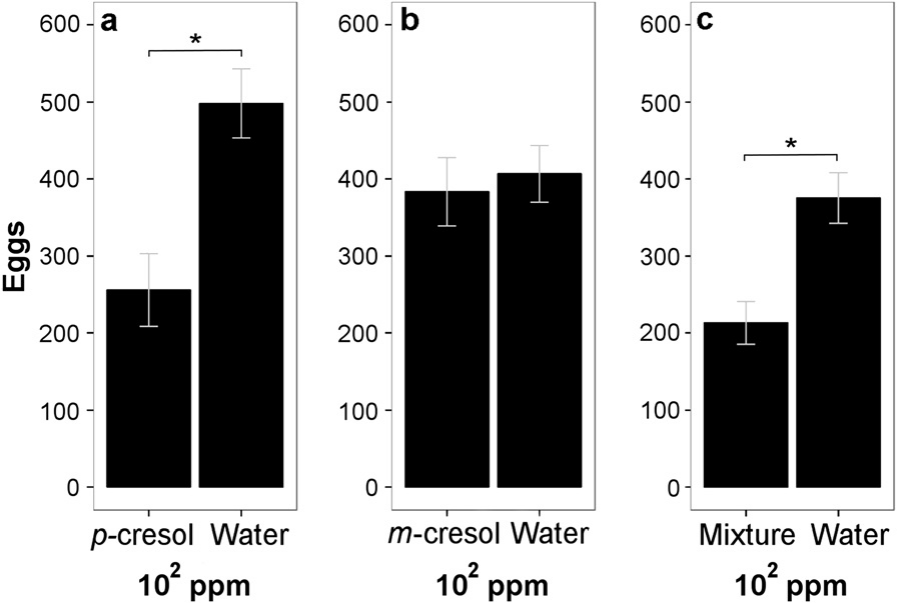
**Oviposition response of *Ae. aegypti *to single odors and their mixture tested against water. a) ***p*-cresol at 10^2^ ppm was deterrent (P = 0.009, paired *t-*test, same data as Figure 4, replotted for comparison, n = 13). **b)***m*-cresol at 10^2^ ppm was not deterrent (P = 0.766, paired *t-*test, same data as Figure 5d, replotted for comparison, n = 13). **c)** The mixture was deterrent (P = 0.008, paired *t-*test, n = 11). Asterisk indicates P < 0.05.

We then tested whether a mixture of both compounds preserves the deterrent effect of *p*-cresol or not. We used a concentration of 10^2^ ppm (50 ppm of both compounds). At this concentration *p*-cresol alone was deterrent (Figure 7a), while oviposition on *m*-cresol did not differ from water (Figure 7b). The mixture received significantly lower number of eggs than water (P = 0.008, paired *t*-test, Figure 7c), indicating that adding *m-*cresol does not diminish the deterrent effect of *p-*cresol.

### Cross-influence of *p*-cresol and *m*-cresol in the same cage

**Figure 8:**
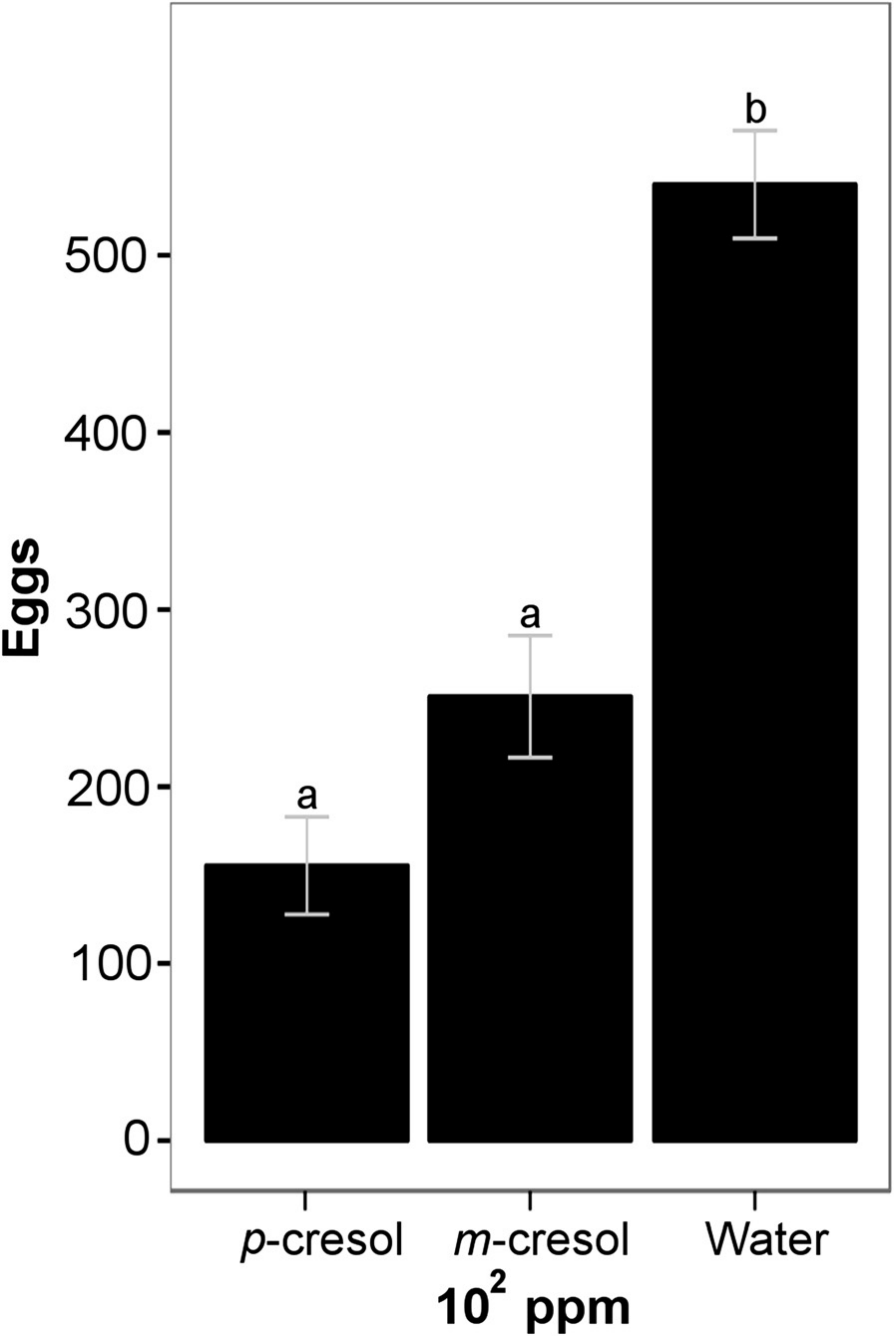
**Oviposition response of *Ae. aegypti *to *m*-cresol, *p*-cresol and water within the same cage. ***m*-cresol at 10^2^ ppm is deterrent (P = 0.001, Pairwise *t*-test with Holm correction) in the presence of *p*-cresol (n = 9). Different letters indicate statistically significant differences of mean.

Given that odorants in the environment influence substrate choice, we asked whether gravid *Ae. aegypti* might behave differently towards *m*-cresol when the isomer *p*-cresol is also present in the air. Therefore, we tested a concentration of 10^2^ ppm *m*-cresol and *p*-cresol against each other and water in the same cage. As reported above, at these concentrations *m-*cresol was not behaviorally active (Figure 7b), while *p-*cresol was deterrent (Figure 7a). When offered side-by-side in the same cage, the group effect was highly significant (P < 0.001, two-way ANOVA), and *p*-cresol received a significantly lower number of eggs than water (P < 0.001, post hoc pairwise *t*-test with Holm correction, Figure 8). Surprisingly, however, *m*-cresol also received a significantly lower number of eggs than water (P = 0.001, post hoc pairwise *t*-test with Holm correction, Figure 8), not significantly different from that laid on *p*-cresol.

## Discussion

Here we demonstrate that *p*-cresol has a dose dependent effect on *Ae. aegypti* oviposition (Figure 4). *p*-cresol deterred oviposition at a broad range of concentrations (10^-8^-10^3^ ppm), and was stimulant only at 10^-10^ ppm. Our results agree with the literature in that *p*-cresol is a strong deterrent for *Ae. aegypti* oviposition, however, they contradict results from an earlier report that *p*-cresol is not deterrent at 100 μg/liter (10^-1^ ppm) [19] and a recently published report that showed the stimulant effect at 4*10^-5^ ppm concentration [21].

The dose dependent effect of *p*-cresol is a common phenomenon with odors that affect mosquito oviposition, in which an odor is stimulant/attractant at low concentrations and deterrent/repellent at higher concentrations. 3-methylindole (Skatole) has a similar dose dependent effect on the oviposition of *Cx. quinquefasciatus *[24], *Toxorhynchites moctezuma* and *Tx. amboinensis *[28]. This effect is also reported for the oviposition pheromone erythro-6-Acetoxy-5-hexadecanolide on *Cx. quinquefasciatus* oviposition [34] and for undecyl decanoate on the oviposition of *Anopheles stephensi *[35]. Plant infusions also have a dose dependent effect in which the mass of plant material and fermentation period play a major role in determining the effect of the infusion on oviposition [6,20,36]. The dose dependent effect of *p*-cresol (alone or in interaction with other compounds) could therefore explain the contradictions in the oviposition effect of Bermuda grass infusions reported earlier [18-20].

On the other hand, the isomer *m*-cresol was not deterrent except at the highest concentration (10^3^ ppm), irrespective of whether concentrations were tested individually (Figure 5) or as a choice across concentrations (Figure 6).

The weak deterrent effect of 10^2^ ppm *m*-cresol when tested in a concentration choice test disappeared when this concentration was tested alone against water. Thus, this weak deterrent effect is not due to the 10^2^ ppm concentration of *m*-cresol *per se*, but rather the result of testing a range of concentrations together. This means that *m*-cresol is a weak deterrent that affects oviposition only at high concentration (10^3^ ppm). We conclude that the two isomers *p-*cresol and *m-*cresol elicit quite dissimilar oviposition responses in *Ae. aegypti* gravid females. This is somewhat surprising, given that the responses to both odorants increase after blood feeding, and that both activate an overlapping set of responses in receptor cells housed in the same sensilla [29]. Whether the deterrent effect of *m*-cresol at high concentrations might be due to cross-talk with a receptor more sensitive to *p*-cresol remains to be tested.

Notably, *p*-cresol, which showed a deterrent effect over a wide range of concentrations when tested individually, showed a deterrent effect only at 10^3^ ppm in concentration choice test. The comparison of these two experiments suggests that not only the odorant emitted from the substrate influenced a mosquito’s behavior, but odorants present in the air of the environment also affected the gravid female’s choice.

However, when *m*-cresol was mixed 1:1 with *p*-cresol at 10^2^ ppm concentration, this mixture was deterrent (Figure 7c), indicating that *m*-cresol had no effect on *p*-cresol.

Finally, we tested *m*-cresol and *p*-cresol at 10^2^ ppm together in the same cage. This concentration was chosen because it is the highest neutral concentration of *m*-cresol, while it has a strong deterrent effect with *p*-cresol. Therefore, we assumed that the presence of *p*-cresol could alter the effect of *m*-cresol at this concentration. We show that *m*-cresol received a significantly lower number of eggs than water when tested together in the same cage with *p*-cresol (Figure 8). This suggests that the response of *Ae. aegypti* gravid females towards a non-deterrent concentration (10^2^ ppm) of *m*-cresol changes when its deterrent isomer *p*-cresol is present in the same cage. The deterrent effect of *m*-cresol that appears only in the presence of *p*-cresol could be due to olfactory generalization. When searching for an oviposition site, mosquitoes land on potential substrates to test the suitability of that substrate to oviposition. If *p*-cresol works only as a short range deterrent rather than repelling mosquitoes from a long distance, a mosquito would have to land on the *p*-cresol vial before deciding that it is not suitable for oviposition. After experiencing such a strong deterrent (10^2^ ppm *p*-cresol), the mosquito might generalize the repellent effect to other, similar, odors, which might resemble that deterrent odor (*m*-cresol), and direct most of the eggs to a less similar odor (water).

Another explanation could be that the mosquitoes smell a mixture of the two odors in the air inside the cage. This diffused odor mixture would have a high concentration of the *p*-cresol component and a lower concentration of the *m*-cresol component around the *p*-cresol cup. Conversely, the odor mixture would have high concentration of the *m*-cresol component and a lower concentration of the *p*-cresol component around the *m*-cresol cup. This means that the mosquitoes might have perceived the odor of *p*-cresol at the *m*-cresol cup. In this case, the gravid female probably also smells a mixture of the two odors around the water cup but at a lower concentration and therefore prefers it over the *p*-cresol and *m*-cresol cups. It would be interesting to test, in future studies, the mixture effect and interaction between *p*-cresol and *m*-cresol at a range of concentrations, and varying relative concentrations, other than 100 ppm.

## Conclusions

*Ae. aegypti* is a vector for yellow fever, dengue and chikungunya diseases. Understanding the factors affecting their oviposition behavior is important to predict their distribution patterns and to develop control programs. Here, we show that *p*-cresol could be used at a very wide range of concentrations to deter oviposition of *Ae. aegypti*. A possible use of this deterrent is in control programs that follow a push and pull strategy, in which a deterrent is used to deter oviposition from one site and an attractant is used to attract oviposition to another site. Here, because at a distance *p-*cresol is likely to occur at low concentrations, and thus be a stimulant, using this substance would reinforce the push-pull strategy. We also showed that *p*-cresol could work as an oviposition stimulant at a very low concentration and therefore could explain the stimulant effect of Bermuda infusions.

We show that *m*-cresol does not have the potential of a deterrent for *Ae. aegypti*. Nevertheless, understanding why *m*-cresol is perceived as a deterrent in the presence of *p*-cresol in adjacent cups could help understand oviposition behavior of *Ae. aegypti* and how this behavior could change in nature when mosquitoes experience complex odors rather than single substances.

Importantly, in this study, we show that oviposition choice is not only determined by the odor of the substrate (as a potential breeding ground for the larvae), but also by the odors present in the surrounding air. Females responded differently to identical stimuli depending on whether other stimuli were present in the same cage or not. *p*-cresol in adjacent pots increased, or even induced, a deterrent effect of other odorants (notably *m*-cresol, Figure 8), or reduced a deterrent effect (on lower concentrations of *p*-cresol, Figure 6). This observation might relate to substrate choice in the wild: not only is the substrate microcosmos important, but also the larger-scale environment may have an important influence on oviposition site choice. This finding adds a note of complexity to pest control schemes: any bait placed in nature will affect nearby baits or natural oviposition sites, and will be affected by them, and these effects can go either towards stronger or towards weaker effects. These air-borne interactions need careful attention in future studies on oviposition-affecting odors. Future studies using semi-field or field assays will need to address how background odors influence the behavioral effect of *p*-cresol, and how the dose-dependent behavioral switch affects mosquito oviposition in nature.

## Competing interests

The authors declare that they have no competing interests.

## Authors’ contributions

AA participated in the design of the study, carried out the experiment, performed the statistical analysis, and drafted the manuscript. GG participated in the design of the study, participated in the statistical analysis, and helped to draft the manuscript. Both authors read and approved the final manuscript.
